# Applying the lessons of implementation science to maximize feasibility and usability in team science intervention development

**DOI:** 10.1017/cts.2021.826

**Published:** 2021-07-22

**Authors:** Betsy Rolland, Felice Resnik, Sarah D. Hohl, LaKaija J. Johnson, Mondira Saha-Muldowney, Jane Mahoney

**Affiliations:** 1 Institute for Clinical and Translational Research, School of Medicine and Public Health, University of Wisconsin-Madison, Madison, WI, USA; 2 Carbone Cancer Center, School of Medicine and Public Health, University of Wisconsin-Madison, Madison, WI, USA; 3 Dissemination & Implementation Launchpad, Institute for Clinical and Translational Research, School of Medicine and Public Health, University of Wisconsin-Madison, Madison, WI, USA; 4 Department of Medicine, University of Wisconsin-Madison, School of Medicine and Public Health, University of Wisconsin-Madison, Madison, WI, USA

**Keywords:** Team science, translational teams, implementation science

## Abstract

The Science of Team Science (SciTS) has generated a substantial body of work detailing characteristics of effective teams. However, that knowledge has not been widely translated into accessible, active, actionable, evidence-based interventions to help translational teams enhance their team functioning and outcomes. Over the past decade, the field of Implementation Science has rapidly developed methods and approaches to increase the translation of biomedical research findings into clinical care, providing a roadmap for mitigating the challenges of developing interventions while maximizing feasibility and utility. Here, we propose an approach to intervention development using constructs from two Implementation Science frameworks, Consolidated Framework for Implementation Research, and Reach, Effectiveness, Adoption, Implementation, and Maintenance, to extend the Wisconsin Interventions for Team Science framework described in Rolland *et al.* 2021. These Implementation Science constructs can help SciTS researchers design, build, test, and disseminate interventions that meet the needs of both *adopters*, the institutional leadership that decides whether to adopt an intervention, and *implementers*, those actually using the intervention. Systematically considering the impact of design decisions on feasibility and usability may lead to the design of interventions that can quickly move from prototype to pilot test to pragmatic trials to assess their impact.

## Introduction

The field of the Science of Team Science (SciTS) has generated a substantial body of work detailing the characteristics of effective teams. However, that knowledge has not been widely translated into accessible, active, actionable, evidence-based interventions for translational teams [1]. A 23 January 2021 search of the Science of Team Science Mendeley library [2], which contains more than 2500 SciTS-related citations, yielded just 8 with the term “intervention” in the title and a mere 97 with the term “intervention” anywhere in their library entry. The limited number of interventions often leaves Translational Teams, defined as those “composed of diverse members who interact, adapt and evolve using established norms and defined roles to address a shared translational objective” [3], receiving vague advice such as “develop trusting relationships,” “hold your collaborators accountable,” and “coordination mechanisms are important,” without giving them tangible, proven ways to achieve those objectives. Each Translational Team may have a unique culture, environment, and set of collaboration challenges, limiting the utility of overly prescriptive advice; however, widely applicable principles of collaboration can inform team-based interventions with the goal of improving team functioning and outcomes.

Team science is not the only field struggling with the questions of translating research findings into practice. For example, fields within health sciences (e.g., public health, nursing, clinical oncology) and beyond (e.g., environmental science) grapple with how to more efficiently apply evidence in real-world settings [4–8]. The field of Implementation Science provides a roadmap for addressing the challenge of developing, testing, and translating SciTS interventions into practice. Biomedical researchers have heard the oft-cited statistics that, on average, it takes 17 years for 14% of original research to reach patients [9]. In a bid to reduce that lag so that innovative, effective health interventions are translated into improved population health more quickly, the National Institutes of Health has invested heavily in the development of the field of implementation science, including funding research and supporting training programs to develop a workforce of Implementation Science specialists. For example, implementation science is a major focus of the National Cancer Institute’s $1.8 billion Cancer Moonshot program [10]; the NIH institutes have also supported mentored training programs such as the Training Institute for Dissemination and Implementation Research in Cancer [11]. Implementation Science is defined by the National Cancer Institute as “the study of methods to promote the adoption and integration of evidence-based practices, interventions, and policies into routine health care and public health settings to improve our impact on population health” [12]. Implementation Science focuses on the process of how to apply scientific discoveries in “real-world” settings, including the design, dissemination, adoption, local tailoring, and evaluation of interventions. To increase adoption, Implementation Science aims to identify how intervention characteristics interact with the target audience in the context in which the intervention is implemented.

Implementation science is uniquely suited to address the research-to-practice gap facing SciTS. Using implementation science frameworks may help overcome this gap by providing translational teams with frameworks, strategies, and constructs to build, design, and test interventions to maximize their effectiveness and feasibility in “real-world” contexts [13, 14]. Leveraging the work that has been done to develop the methods, tools, and approaches of Implementation Science, including two frameworks described below, we can potentially dramatically increase the number of interventions that are available to Translational Teams to help them improve their team functioning. In this paper, we (1) describe the four-phase process of the Wisconsin Intervention for Team Science (WITS); (2) describe and provide examples of how two implementation science frameworks informed design and testing of the WITS; and (3) discuss the use of a proposed rubric to help guide SciTS researchers and practitioners as they develop interventions focused on feasibility and usability.

### The Intervention Development Process: The Wisconsin Interventions for Team Science (WITS) Framework

**Fig. 1:**
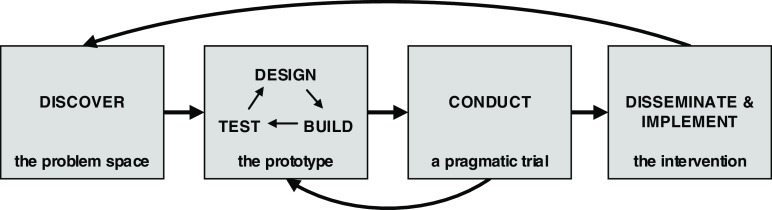
Wisconsin Interventions for Team Science Framework: A Four-Phase Approach to TeamScience Intervention Development (Rolland et al. 2021) [1].

Numerous fields, including health services research, Human Centered Design and Systems Engineering, have proposed process models for developing interventions. The WITS Framework (Fig. 1) was developed with the goal of increasing the rigorous translation of SciTS research into practice [1]. We propose that the WITS framework can equip SciTS researchers and practitioners engaged in studying and facilitating translational team science with a practical way to conceptualize the iterative process of intervention development, testing, and widespread adoption and use. It extends the Discover, Design/Build, and Test framework proposed by Lyon *et al.* [15] and the Diffusion-Dissemination-Implementation Continuum [16] to include broad dissemination and implementation in practice. Further, the WITS Framework proposes activities and general evaluation criteria for moving from one step to the next, gathering evidence along the way to support the intervention’s effectiveness. Each step of this process requires attention to design decisions that can be informed by Implementation Science constructs.

Phase 1 consists of discovering the problem space, including the team-based challenge, the end users, and the context of those users. Intervention developers should identify and assess the characteristics, needs, and challenges experienced by the end users and identify potential strategies to meet the identified translational team needs.

In Phase 2, intervention developers engage in designing, building, and testing the prototype. Designing requires definition of the intervention components, deciding the form of the intervention, who will deliver or implement it, and how success will be measured, as well as how the intervention will be disseminated and what kinds of training the implementers of the intervention will need. Finally, a rigorous strategy for testing feasibility must be laid out. In the Build activities of Phase 2, the intervention developers create the prototype intervention, while the Test activities include testing the prototype with stakeholders, including the potential end users, implementers, and adopters (i.e. university centers, programs, or departments where the intervention will be provided).

Phase 3 is when the intervention is tested more rigorously with representative teams to assess the effectiveness of the intervention in real-world settings.

Finally, Phase 4 is when the intervention is disseminated and implemented more broadly, testing the effectiveness “in the wild,” or beyond the controlled conditions of the pragmatic trial. This dissemination may include posting the intervention materials online where anyone can download and implement them with their teams or it may involve training a team of facilitators to deliver the intervention without the engagement of the original intervention development team.

### Lessons from Implementation Science

At each of these steps, intervention developers must make decisions about how to translate SciTS findings into interventions that are appropriate for Translational Teams and that these teams are likely to use. Two Implementation Science frameworks, Consolidated Framework for Implementation Research (CFIR) [17] and Reach, Effectiveness, Adoption, Implementation, and Maintenance (RE-AIM) [18], can help us think about those decisions in a way that has proven effective for translating health research into practice. In this section, we introduce the key constructs of CFIR and RE-AIM, with a focus on using these constructs to assess or predict how feasible and usable an intervention might be. In the Implementation Science literature, feasibility refers to the “[e]xtent to which the intervention can be successfully used within a given setting” [12], while usability focuses on how easy the intervention is to actually use [19]. To help illustrate each of the CFIR and RE-AIM constructs, we briefly describe some of the design decisions we made while adapting and implementing the original Collaboration Planning framework [20, 21] as an intervention. More details of this adaptation and implementation were described in a previous publication [22].

#### The consolidated framework for implementation research (CFIR) framework

The CFIR framework is an adaptable framework focused on examining complex contextual factors (i.e. determinants) that affect implementation and effectiveness of an intervention in a particular setting. The CFIR incorporates 39 constructs organized across 5 domains that can be selected based on fit and used to assess contextual factors. We propose that if the CFIR is meaningfully utilized when developing team science interventions, it will help to develop deeper understanding of the contextual factors affecting the implementation and delivery of an intervention, allowing for design of an intervention that will be feasible to implement in the setting for which it is intended, and feasible to tailor for use in other settings.

1. *Intervention Characteristics* focuses on understanding the attributes of the intervention that may impact the success of implementation in a specific organization. This domain includes eight constructs, including adaptability. Aspects to consider include which elements can be adapted or tailored to meet the needs of the organization.

*Collaboration planning design decisions*

We invested substantial time in understanding how the Intervention Characteristics might help translational teams address a key challenge: launching with effective team processes. We quickly honed in on Collaboration Planning as a framework whose characteristics helped us achieve that goal. It required a relatively low time commitment, drew from the SciTS evidence base, and was an exercise whose point and outcomes teams could easily understand.

1. *Outer setting* considers how factors such as the cultural, social, and economic context of the organization may impact implementation using four constructs. It could include the assessment of the construct of external policies and incentives by examining external guidelines that impact the organization and intervention.

*Collaboration planning design decisions*

As we thought about our Outer setting, we considered how it would benefit UW-ICTR within the CTSA consortium, as well as how implementing the intervention could help us meet the requirements of the CTSA funding opportunity announcement.

1. *Inner Setting* helps to examine characteristics of the organization or unit implementing the intervention, including assessment of aspects such as structural characteristics, culture, and readiness for implementation. This assessment could mean gaining a better understanding of a translational team’s values and norms and how to use this knowledge to better implement the intervention.

*Collaboration planning design decisions*

Critical to designing for our Inner Setting was to consider our internal goals and how the intervention might help us meet those, as well as how we would measure goal achievement for our program and for our individual teams. It was also critical to consider the length of time that a translational team would consider reasonable for receiving an intervention, as well as any time or capacity constraints on the unit delivering the intervention. We are still working on identifying short-, medium-, and long-term measures of outputs, outcomes, and translational science benefits [23]; many of these measures require substantial time investment from translational teams and our Team Science core.

1. *Characteristics of Individuals* highlights how the individuals who are involved in the organization may impact the implementation of the intervention. This domain considers the interplay between individuals and the translational teams of which they are members. Here, we are considering dynamic constructs including individuals’ knowledge and beliefs about the intervention, self-efficacy, and personal attributes that may affect implementation.

*Collaboration planning design decisions*

We kept at the forefront the specific type of translational teams for which we were designing, namely, UW-ICTR pilot teams and the individuals who made up those team. The Characteristics of Individuals helped us think about who our pilot awardees were and how the intervention might interact with their existing concepts of teamwork, their other responsibilities to the university, and their experience working with one another.

1. *Process* refers to the implementation approach. This domain provides a broad view of the stages of implementation, which are iterative: Engaging, Executing, Planning, and Reflecting and Evaluating. It could include engaging with stakeholders and creating the implementation plan for the team science intervention.

*Collaboration planning decisions*

Our Process for designing the intervention relied heavily on our own experience working with UW-ICTR pilot teams (and other nascent scientific teams) to create an intervention that fit within researchers’ workflows. We adapted the original framework, tested it with real teams, and have used data from an evaluator-observer and from post-session surveys of session participants to rapidly iterate the intervention components.

#### The reach, effectiveness, adoption, implementation, and maintenance (RE-AIM) framework

The RE-AIM framework is one of the most widely used tools for thinking about implementation science and evaluating the implementation of an intervention [18]. We propose that it can be useful, too, in the context of thinking about team science interventions and can help team science researchers think about how to design, develop, evaluate, and scale-up effective team science interventions.

1. *Reach* helps us consider whether we are getting the intervention to those who need it. Reach focuses on questions of how you are defining the audience (end user) for this intervention, how you will measure reach for each audience segment/type, what would be considered successful reach for this intervention and what are barriers and facilitators for reaching different audiences.

*Collaboration planning decisions*

The concept of *Reach* led us to consider what we knew about UW-ICTR pilot teams that could help us think about successful engagement for the Collaboration Planning intervention, as well as the ways we needed to contact them and market the sessions to engage those teams in the intervention. Pilot studies are generally modest in size and funding, with minimal dedicated personnel effort, so our intervention needed to accommodate those constraints and not require teams to devote substantial time to participating.

1. *Effectiveness* focuses on how we know if the intervention is working. Here, we can consider how to define the outputs, outcomes, and impact of the intervention, how to measure effectiveness for the audience defined in Reach, and what effectiveness would look like beyond testing in a controlled environment.

*Collaboration planning decisions*

We developed success metrics to measure Effectiveness, initially focusing on measures of engagement and value in the feasibility testing phase. As we gear up for a pragmatic trial of this evidence-informed intervention, we are defining broader metrics of success that might indicate enhanced team processes post-session such as increased coordination mechanisms or team satisfaction.

1. *Adoption* raises questions around the organization’s willingness to take up the intervention, and the support that is provided for implementation of the intervention (i.e., buy-in at the top). The “Adopter” is who—at the team or organizational level—decides to take up the intervention. Here, we consider the supports that are provided for those who facilitate and deliver the intervention (the “Implementers”), and the kinds of local support that are needed to ensure the intervention is implemented effectively and sustainably, and whether the organization is even prepared for the intervention. Also relevant are the barriers and facilitators to adoption by different organizations, with a focus toward designing the intervention to maximize their ease of adoption.

*Collaboration planning design decisions*

We worked directly with the UW-ICTR pilot award administrators and Workforce Development team, who served as the Adopters for this intervention, so expended minimal effort to convince them to support Collaboration Planning for the pilot teams. However, as we move forward to broader testing, the key questions of Adoption will center around how to engage with decision-makers across the CTSA consortium and beyond.

1. *Implementation* focuses on ensuring the intervention is delivered properly by the Implementers who deliver the intervention to end users. The Implementation construct requires considering barriers and facilitators to implementation by different organizations, what kinds of training are needed to make sure the intervention is delivered as intended (i.e., fidelity), how fidelity will be measured, whether implementers will need an official guidebook or training to be able to deliver the intervention, and whether and how much adaptation to the local context is necessary and appropriate. In addition, it is worthwhile to consider whether different audiences might require variations of the intervention.

*Collaboration planning decisions*

Our focus in designing our adaption was to deliver a lightweight, facilitated, 90-min session with pilot teams to help them launch with effective team processes. Since the initial feasibility test, we have developed a comprehensive Facilitators Guide and 3-hour Facilitator Training to ensure a rigorous *Implementation* of the intervention with high feasibility.

1. *Maintenance* refers to the ways in which Implementers should incorporate the intervention into regular practice so it continues to be delivered over the long term. It could include questions like how can you make sure the behaviors you have encouraged in your intervention are maintained over the life of the team, or at the institutional level, what kinds of infrastructural support need to be built to sustain intervention delivery over time.

*Collaboration planning decisions*

We frankly did not think much about *Maintenance* in the first iteration of Collaboration Planning but have recently begun to think about how we can perhaps offer mid-year or end-of-year check-ins or tune-ups with teams who have undergone Collaboration Planning. The question of how to design for maintenance at the institutional level will get more challenging as the intervention is disseminated more broadly across many different kinds of institutions.

#### The Application of CFIR and RE-AIM constructs to Develop Team Science Interventions

As Team Science Intervention developers proceed through the phases of intervention development, the CFIR and RE-AIM constructs can serve as a guide for thinking more broadly about the adopters, the implementers, the target audience, the organizational context, and the process by which the intervention will be implemented. By designing with adoption by other organizations in mind using CFIR and RE-AIM, an intervention can be designed to maximize the likelihood of scale-up and impact in broad practice. Table 1 provides a rubric for assessing how likely an intervention is to be feasible and usable, with criteria for each of the CFIR and RE-AIM constructs that are likely to lead to low or high feasibility and usability. We envision this rubric being used by intervention developers during the process of intervention development, as well as potentially being used to develop evaluation criteria for the intervention itself. It is also our goal to continue to integrate the constructs of this model into the WITS Framework, providing additional guidance and support for intervention developers.

**Table 1.** WITS rubric for intervention development

**CFIR Construct**	**Criteria likely to lead to low feasibility and usability of the intervention**	**Criteria likely to lead to high feasibility and usability of the intervention**
Intervention characteristics	No analysis has been done of the “fit” between the intervention and the organization where it is being implemented	Characteristics of the intervention have been assessed in terms of their “fit” with the organization where it is being implemented
Outer setting	Implementation of the intervention serves no external purpose for the organization, does not enhance its status or relationships in any way	Implementation of the intervention is likely to benefit the organization externally (e.g., meeting a funding agency’s requirements, competitive advantage)
Inner setting	Implementation of the intervention does not meet internal organizational needs, does not fit with the culture and norms of the organization	Implementation of the intervention helps the organization or the implementers meet an internal goal. It fits with the culture and norms of the organization. The implementers have the capabilities to implement the intervention, and the organization is ready for this change.
Characteristics of individuals	The outcomes of the intervention do not align with the personal goals of the members of the Translational Team. The team members do not have the skills, knowledge, and attitudes to adopt the intervention.	The intervention aligns with the goals of individual members of the Translational Team. Those members are ready for change, and the intervention fits with their own values.
Process	The intervention plan is haphazard and does not align with the team’s workflow.	The plan for implementing the intervention is well considered and fits within the organizational workflow.
**RE-AIM Construct**		
Reach	Poor definition of the target audience and their challenges	Co-design of intervention with target audience(s) (translational team). Team’s confidence in the intervention’s ability to address their challenges.
Effectiveness	No theoretical basis for the intervention’s effectiveness. No measurable definition of success.	Use of literature from SciTS, behavior change theory, and pedagogical theories
Adoption	Institutional leadership is not interested or willing to invest resources to support implementation of the intervention. No ability to communicate the value of the intervention to the institution.	Institutional leadership is willing to invest resources in adoption. They understand how the elimination of the challenge/barrier will benefit the institution.
Implementation	No rigorous guide for implementation. Intervention is challenging to implement and requires large investment of time or other resources. Intervention must be delivered exactly as described no matter the context.	Easy-to-replicate delivery of intervention and/or trained facilitators. Measurable fidelity metrics. Flexible in its tailoring to new contexts while ensuring the fidelity of delivery of the intervention’s key elements.
Maintenance	At team level: Predicted effect of the intervention is short-lived and not maintained over time. There is no “tune-up” or “check-in” plan with the intervention. No criteria for short- and long-term intervention outcomes for the teams. At organizational level: Institutional leadership does not support continued use of the intervention; use is not maintained over time.	At team level: Clear maintenance plan for teams to integrate the new knowledge, skills, behaviors into daily workflow. Ability of the teams themselves to measure their progress. At organization level: Organization finds continued value in use of the intervention over time and has plan to continue use of the intervention.

WITS, Wisconsin interventions for team science; CFIR, the consolidated framework for implementation research; RE-AIM, the reach, effectiveness, adoption, implementation, and maintenance framework; SciTS, Science of Team Science.

### Future Work

The constructs described here have been used by the UW-ICTR team science and Dissemination & Implementation Launchpad^TM^ teams to develop our Collaboration Planning intervention [22] and also draw from our collective experience with application of dissemination and implementation frameworks in other fields (e.g., tobacco cessation for cancer patients, falls prevention, evidence-based self-management interventions) [24–30]. However, this approach needs its own rigorous testing, and we invite SciTS researchers to consider how the process can be used to develop additional interventions to solve the challenges of collaboration for Translational Teams. One place the field might start is by focusing on developing interventions to tackle the seven challenges of team science identified by the 2015 National Research Council report on Enhancing the Effectiveness of Team Science [31], which include:

1. High diversity of membership
2. Deep knowledge integration
3. Large team size
4. Goal misalignment with other teams
5. Permeable boundaries
6. Geographic dispersion
7. High task interdependence

What would interventions to address these challenges look like? How could they be designed to be feasible and useful in multiple Translational Team contexts and reach the intended audiences? How could interventions be designed to maximize their potential adoption by different organizations? How would we rigorously measure and test the outcomes of these interventions? How can the CTSA program help scale-up these interventions across CTSA hubs and beyond? How can we use this intervention development process to increase our understanding of the unique challenges and opportunities of Translational Teams?

We also encourage teams developing interventions to report more on the process they follow to develop their interventions. In the future, we hope to develop a brief reporting guideline similar to the Consolidated Standards of Reporting Trials (CONSORT) statement for reporting clinical trials [32] or the Standards for Reporting Implementation Studies (StaRI) Statement [33]. Reporting on the design process, as well as the ways in which the intervention was tested and the resulting effectiveness data, allows other SciTS researchers to build upon those findings and also allows adopters and implementers to assess the feasibility and usability of the intervention in their specific context. We also are currently developing more detailed guidance for integrating the constructs described in this paper into the full intervention development process, from design to widespread dissemination and implementation and hope to share that work soon. Finally, we hope that increased testing and reporting of results for intervention dissemination and implementation will help the SciTS field develop more innovative methods for testing our work in the real world.

## Conclusion

The feasibility and usability of interventions to facilitate the formation, management, and leadership of high-impact Translational Teams can be enhanced by designing interventions that are specifically targeted to meet the unique challenges and opportunities of Translational Teams, including their focus on moving innovations from discovery to the communities we serve, their inclusion of multiple disciplines, and their dynamic membership over the course of a project. By using CFIR to design a team science intervention to maximize its potential reach, adoption, sustainability, implementation, and maintenance (thinking in RE-AIM terms), we are designing to maximize feasibility and use. These Implementation Science constructs can help SciTS researchers design, build, test, and disseminate interventions to improve team functioning that meet the needs of both adopters, the institutional leadership that decides whether to adopt an intervention, and implementers, those actually using the intervention.

The SciTS has developed substantial knowledge about the characteristics and approaches that lead to high-functioning teams. Yet, Translational Teams still struggle to work together effectively, in part because that SciTS research remains hidden in academic publications, rarely translated into feasible, usable, evidence-based interventions to help Translational Teams. Developing, testing, and adhering to rigorous approaches to translate the field’s findings into interventions can increase the likelihood of those interventions being feasible and usable for Translational Teams not only in one setting but also across multiple settings. The field of Implementation Science has paved the way for these approaches. By adapting and adopting constructs from Implementation Science frameworks such as CFIR and RE-AIM to our team science intervention design process, we can more rapidly advance both the SciTS field and our impact on solving the complex scientific problems faced by Translational Teams.
